# Carbohydrate-Based Chiral Ligands for the Enantioselective Addition of Diethylzinc to Aldehydes

**DOI:** 10.3390/ph18081088

**Published:** 2025-07-23

**Authors:** F. Javier López-Delgado, Daniele Lo Re, F. Franco, J. A. Tamayo

**Affiliations:** Department of Medicinal and Organic Chemistry, School of Pharmacy, University of Granada, Campus de Cartuja, 18071 Granada, Spain; javier.lopez@destinagenomics.com (F.J.L.-D.); da.lore@ugr.es (D.L.R.)

**Keywords:** carbohydrates, asymmetric catalysis, titanium, zinc

## Abstract

**Background**: Carbohydrate-derived chiral ligands are promising tools in asymmetric catalysis due to their structural diversity, chirality, and availability. However, ligands based on galactose or sorbose have been scarcely explored in the enantioselective addition of dialkylzinc reagents to aldehydes. **Methods**: A series of chiral diols and β-amino alcohols was synthesized from methyl D-glucopyranoside, methyl D-galactopyranoside, and D-fructose. These ligands were tested in the titanium tetraisopropoxide-promoted enantioselective addition of diethylzinc to aromatic and aliphatic aldehydes. **Results**: Several ligands, particularly those with a D-fructopyranose backbone, exhibited excellent catalytic activity, with conversion rates up to 100% and enantioselectivities up to 96% *ee*. Notably, this study reports for the first time the use of β-amino alcohols derived from fructose and sorbose in this transformation. **Conclusions**: Carbohydrate-based ligands represent effective, inexpensive, and structurally versatile scaffolds for developing highly enantioselective catalysts, expanding the utility of sugars in asymmetric organometallic reactions.

## 1. Introduction

The enantioselective addition of dialkylzinc compounds to molecules containing prochiral carbonyl groups is a powerful tool for obtaining enantiopure secondary or tertiary alcohols [1,2,3,4,5]. Since the early work of Oguni, Noyori, and Soai [6,7,8,9,10,11], where they reported the asymmetric addition of diethylzinc to carbonyl compounds in the presence of catalytic amounts of a β-amino alcohol, research in this field has grown exponentially [12,13,14].

It has been well established that Ti(IV) complexes efficiently catalyze the asymmetric addition of dialkylzinc reagents to aldehydes, affording high yields and excellent enantioselectivities [15,16]. A variety of chiral ligands have been successfully employed in this transformation, including chiral diols [17,18], BINOL derivatives [19,20,21], disulfonamides [22,23], and N-sulfonylated amino alcohols [23,24,25].

The growing demand for efficient and stereoselective catalysts in environmentally friendly organic synthesis highlighted the potential of carbohydrate-based organocatalysts as chiral ligands. Carbohydrates are abundant, inexpensive, chiral, and biocompatible natural products that offer a versatile platform for catalyst development. Their rigid pyranose and furanose rings provide structural stability and precise stereochemical control, while their multiple hydroxyl groups serve as functional binding sites and can be easily modified to influence catalytic behavior and selectivity. The non-racemic nature, presence of multiple stereogenic centers, and lone electron pairs on ring oxygen atoms further enhance their utility in fine-tuning catalytic performance. Given these features—particularly their low cost, built-in chiral information, and the wide variety of naturally available chiral substrates—carbohydrates are ideal candidates for the development of chiral ligands in asymmetric synthesis and chiral auxiliaries in organocatalysis [26,27,28].

Carbohydrate-based ligands have proven effective in a wide range of asymmetric transformations [26,27,28] including additions of diorganozinc reagents to aldehydes [29,30,31,32,33,34,35,36].

**Figure 1:**
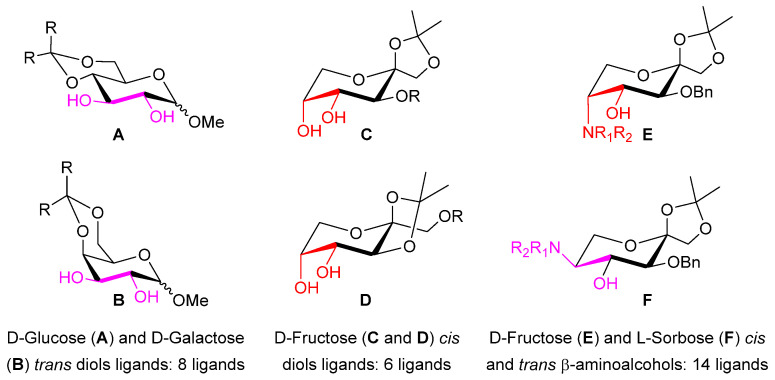
General structures of the ligands described in this paper.

Interestingly, in the asymmetric addition of diethylzinc to carbonyl compounds, only a few examples involving glucose [29,30,31,32,33] have been reported, and only one example using fructose [34] is known. Moreover, no studies to date employed ligands with galactose or sorbose configuration in the enantioselective alkylation of carbonyl compounds with dialkylzinc reagents. This gap is particularly notable given the structural diversity and chiral richness of carbohydrates, which make them ideal scaffolds for designing novel ligand architectures. The present work seeks to address this underexplored area by investigating carbohydrate-based ligands in the asymmetric alkylation of carbonyl compounds, thereby expanding the scope of carbohydrate applications in asymmetric catalysis. As part of our ongoing research in carbohydrate chemistry, and building on our experience in the synthesis of carbohydrate derivatives [37,38,39], we sought to explore the potential of sugar-based scaffolds as chiral ligands in catalytic asymmetric transformations. We initially prepared diols derived from methyl D-glucopyranoside and methyl D-galactopyranoside, which were evaluated as ligands in asymmetric catalysis. Subsequently, structurally related diols based on D-fructose were synthesized and tested under similar conditions. Finally, in the search for ligands with improved catalytic performance, we turned our attention to aminohydroxyfructose derivatives. To the best of our knowledge, these ligands remain largely unexplored in the context of asymmetric catalysis, despite their promising structural features. We report the synthesis and evaluation of carbohydrate-derived chiral ligands, including diols from methyl D-glucopyranoside (Figure 1A), methyl D-galactopyranoside (Figure 1B), and D-fructose (Figure 1C,D), as well as amino alcohols from D-fructose and with L-sorbose configuration (Figure 1E,F). These ligands were tested in the enantioselective addition of diethylzinc to aromatic and aliphatic aldehydes. Their straightforward synthesis from inexpensive, readily available sugars underscores their practical value. Notably, to the best of our knowledge, this is the first report describing the use of β-amino alcohols derived from fructose and sorbose as chiral ligands in the asymmetric alkylation of carbonyl compounds with dialkylzinc reagents. 

## 2. Results and Discussion

Since our objective was to synthesize chiral ligands derived from simple carbohydrates, we began by exploring diols obtained from *D*-glucose and *D*-galactose, which are commercially available and relatively inexpensive starting materials.

**Figure 2:**
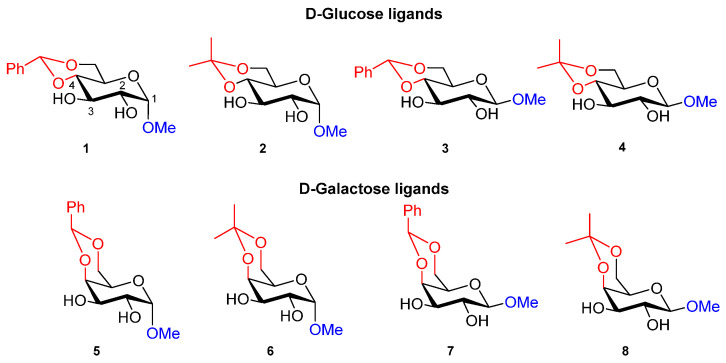
D-glucose and D-galactose-based ligands tested in this study.

In this manner, eight carbohydrate-derived diol ligands (Figure 2. **1**–**8**) previously described in literature were readily synthesized using previously reported procedures [40,41,42,43,44,45,46] (see supporting info) and evaluated with the enantioselective addition of diethylzinc to benzaldehyde. We investigated the influence of the *trans*-diol configuration at C2 and C3, in combination with various protecting groups at C4 and C6, as well as different stereochemistry at C1 and C4.

### 2.1. Optimization of Reactions Conditions in the Enantioselective Addition of Diethylzinc to Benzaldehyde

The reaction conditions for the addition of diethylzinc to benzaldehyde were optimized based on the conditions reported by Bauer et al. [29] for the asymmetric addition of diethylzinc to aldehydes using similar ligands derived from α-D-methylglucopyranoside.

For this reason, ligand **1** was used for the optimization, in which parameters such as solvent, temperature, ligand concentration, the amount of Et_2_Zn, and the amount of Ti(O*^i^*Pr)_4_ were studied (Table 1).

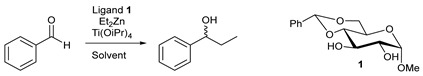



**Table 1:** Optimization of diethylzinc addition to benzaldehyde catalyzed by ligand **1** ^a^.

Entry	Ligand (%)	Ti(O*^i^*Pr)_4_ (eq.)	Et_2_Zn ^b^ (eq.)	Solvent	T (°C)	Conversion (%) ^d^	*ee* (%) ^e^
1	**-**	1.4	3	Hexane ^c^	0	100	0
2	**10**	1.4	3	Hexane ^c^	0	91	32 (*S*)
3	**20**	1.4	3	Hexane ^c^	0	86	56 (*S*)
4	**30**	1.4	3	Hexane ^c^	0	52	55 (*S*)
5	**40**	1.4	3	Hexane ^c^	0	20	45 (*S*)
6	**20**	-	3	Hexane ^c^	0	3	0
7	**20**	0.8	3	Hexane ^c^	0	42	47 (*S*)
8	**20**	1	3	Hexane ^c^	0	81	52 (*S*)
9	**20**	1.2	3	Hexane ^c^	0	72	55 (*S*)
10	**20**	1.6	3	Hexane ^c^	0	88	48 (*S*)
11	**20**	2	3	Hexane ^c^	0	98	46 (*S*)
12	**20**	1.4	3	Hexane ^c^	25	100	38 (*S*)
13	**20**	1.4	3	Hexane ^c^	−28	60	44 (*S*)
14	**20**	1.4	3	Hexane ^c^	−76	12	46 (*S*)
15	**20**	1.4	3	Toluene ^c^	0	70	53 (*S*)
16	**20**	1.4	3	DCM ^c^	0	70	49 (*S*)
17	**20**	1.4	2	Hexane ^c^	0	65	54 (*S*)
18	**20**	1.4	2.5	Hexane ^c^	0	98	52 (*S*)

^a^ 0.25 mmol of benzaldehyde, reaction time 3 h. ^b^ The commercial Et_2_Zn used is a 1M solution in hexane. ^c^ 0.25 mL. ^d^ Determined by GC using a Supelco α-DEX 325 column, helium flow: 0.8 mL/min, temperature: isothermal at 110 °C. ^e^ Absolute configuration assigned by comparing the optical rotation of the purified product with literature data.

The enantioselective addition of Et_2_Zn to benzaldehyde was initially investigated by varying the molar ratio of the ligand (entry 1–5). In the absence of a ligand, Ti(O*^i^*Pr)_4_ appeared to activate Et_2_Zn, generating a catalytic species capable of promoting the addition reaction. However, this transformation proceeded without stereocontrol, yielding the corresponding racemic secondary alcohol (entry 1). When the ligand was introduced at loadings between 10% and 40% (entry 2–5), a degree of enantioselectivity was observed (*ee* 32–45%). On other hand, as the ligand concentration increased, the conversion rate progressively declined, suggesting a possible inhibitory effect at higher ligand loadings.

To further elucidate the role of Ti(O*^i^*Pr)_4_, we examined its influence on both the yield and enantioselectivity of the reaction in the presence of ligand **1**. As expected, Ti(OiPr)_4_ was essential, as no enantioselective addition of Et_2_Zn to benzaldehyde occurred in its absence (entry 6). The reaction tolerated an increase in Ti(O*^i^*Pr)_4_ concentration of up to 1.4 equivalents, beyond which a slight decrease in enantioselectivity was observed (entry 10 and 11). Importantly, the reaction was highly sensitive to temperature variations. Attempts to modify the temperature from 0 °C were unsuccessful. Elevating the temperature to 25 °C resulted in a higher conversion rate but led to a significant erosion of enantioselectivity (entry 12). Conversely, decreasing the temperature to −28 °C or −76 °C led to a drastic reduction in conversion, while enantioselectivity remained moderate (entries 13 and 14). We also investigated the effect of solvent choice on the reaction outcome. Hexane was superior both in conversion rate and enantioselectivity compared with toluene and DCM (entries 16 and 17). On other hand, enantioselectivity remained largely unaffected by changes in Et_2_Zn stoichiometry, with the highest enantiomeric excess obtained at 3 equivalents of Et_2_Zn. The highest conversion rate was achieved with 2.5 equivalents of Et_2_Zn, although this condition resulted in a slightly lower enantiomeric excess than the reaction conducted with 3 equivalents. Therefore, prioritizing enantioselectivity over conversion, we selected 3 equivalents of Et_2_Zn as the optimal stoichiometry under the optimized reaction conditions with ligand **1**.

### 2.2. Evaluation of Chiral Diol Ligands Based on α-, β-D-Methylglucopyranoside and α-, β-D-Methylgalactopyranoside in the Enantioselective Addition of Diethylzinc to Benzaldehyde

Under the previously established conditions (ligand 20%, Ti(O*^i^*Pr)_4_ 1.4 eq, Et_2_Zn 3eq, 0 °C, Hexane), the ability of the synthesized D-glucose and D-galactose ligands (**1**–**8**) to induce the enantioselective addition of diethylzinc to benzaldehyde, was evaluated (Table 2).

The glucose derivatives **1**–**4** exhibited good conversion rates (75–90%) while maintaining moderate enantioselectivity (35–56% *ee*). In comparison, the galactose derivatives **5**–**8** also achieved high conversion (80–89%), but their enantioselectivity was noticeably lower (4–40% *ee*). Acetonide at C-4 and C-6 had generally a significant impact, since benzylidene derivatives led to higher enantiomeric excesses than their isopropylidene counterparts. In the glucose series (ligands **1**–**4**), the α-methyl derivatives consistently outperformed their β-counterparts in terms of enantioselectivity. Interestingly, the opposite effect was observed in the galactose series (ligands **5**–**8**), where the β-anomers perform slightly better than their α-counterparts, while α-methyl galactopyranoside derivatives, **5** and **6**, gave the *R* enantiomer as the major product, setting them apart from the rest of the series.

### 2.3. Evaluation of Chiral Diol Ligands Based on D-Fructose in the Enantioselective Addition of Diethylzinc to Benzaldehyde

**Figure 3:**
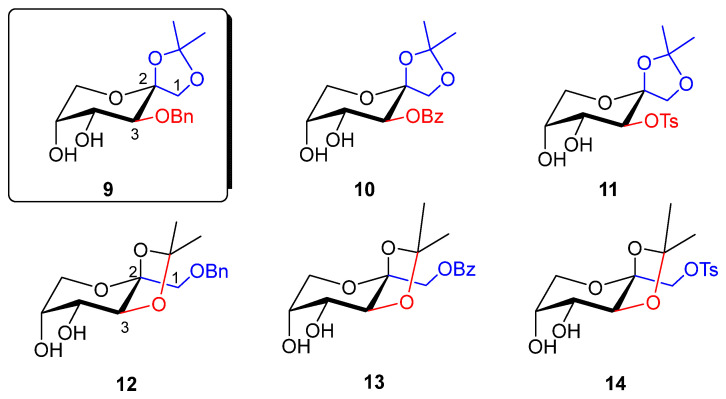
D-fructose ligands featuring 1,2-*cis*-diol moieties.

Since it was postulated that the diol motif in glucose and galactose ligands **1**–**8** was important for enantioselectivity, we wondered if diols derived from D-fructose would influence the enantioselectivity of the Et_2_Zn addition. To test this idea, we synthesized pyranose D-fructose derivatives, bearing a *cis* diol at C-4 and C-5 and strategically incorporating an acetonide protecting group at either C-1/C-2 or C-2/C-3. These ligands (**9**–**14**) (Figure 3), previously described in literature, were prepared following established literature procedures [47,48,49,50,51,52,53,54,55] (see Supporting Info) and tested in the enantioselective addition of Et_2_Zn to benzaldehyde by applying the optimized conditions used for ligands **1**–**8**. The results are presented in Table 3.

Ligands **9** and **10**, bearing an acetonide at C1 and C2 and a benzyl and benzoyl protecting group at C-3, respectively, exhibited excellent catalytic activity, achieving complete conversion with moderate to high levels of enantioselectivity. In contrast, ligand **11**, which features a tosylate group at C-3, displays a high conversion rate (80%) together with a dramatic drop in terms of the enantioselectivity (6% *ee*), suggesting that the substituent at C-3 is crucial for the ligand’s ability to efficiently control the stereochemical outcome of the reaction.

Along the same lines, ligands **12**, **13**, and **14**, bearing an acetonide at C-1 and C-3, displayed high conversion rates (90–99%), together with an important loss in enantioselectivity (11–15% *ee*), highlighting the pivotal role of C-3 in enantio-induction, reinforcing the idea that its functionalization and spatial arrangement are key to achieving effective chiral discrimination. Notably, across all tested ligands, the reaction predominantly led to the formation of the *S* enantiomer, indicating a consistent stereochemical preference within this system.

### 2.4. Synthesis and Evaluation of β-Amino Alcohol Ligands Based on D-Fructose in the Enantioselective Addition of Diethylzinc to Benzaldehyde

Based on these results, we proceeded to prepare fructose-derived ligands bearing both *cis*- and *trans*-β-amino alcohol moieties, which represent one of the most extensively studied classes of chiral ligands and auxiliaries [14].

**Scheme 1:**
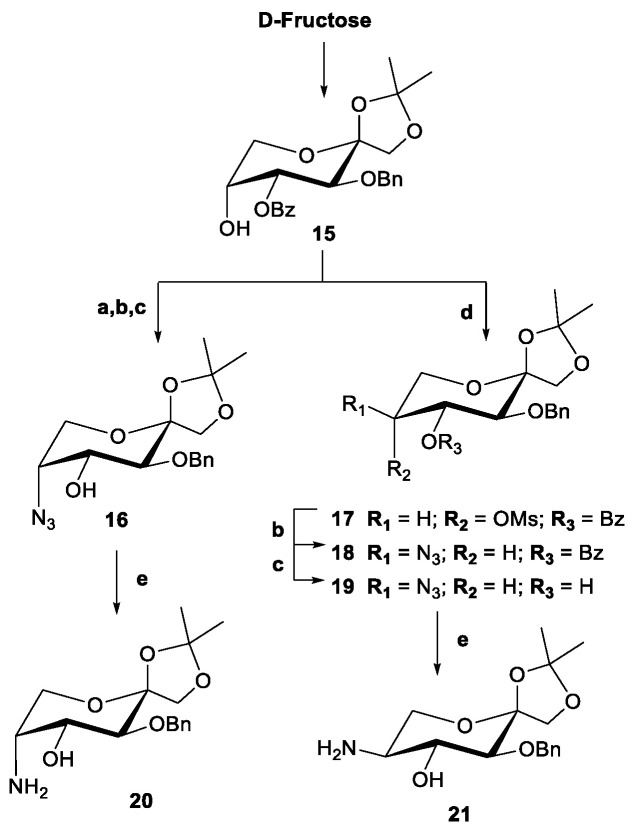
Synthesis of aminoalcohol ligand intermediate **20** and **21**. Reagent and conditions: (**a**) I_2_, Ph_3_P, imidazole, toluene. (**b**) NaN_3_, dry DMF, 110 °C. (**c**) MeONa 1M in MeOH. (**d**) ClMs, Py, 0 °C. (**e**) H_2_, Pd/C, MeOH.

Novel ligands **22**–**27** were synthesized from key intermediate **20** and novel ligands **28**–**33** from key intermediate **21** [56] (Scheme 1). Both intermediates **20** and **21** were obtained from a common precursor, the well-known 4-*O*-benzoyl-3-*O*-benzyl-1,2-*O*-isopropylidene-β-D-fructopyranose **15** [37] synthesized in four steps from D-fructose [48]. Key intermediated β-amino alcohol **20** was achieved from azide **16** [48,57,58]. Catalytic hydrogenation of **16** with Pd/C yielded **20** in high yield. On the other hand, key intermediate β-amino alcohol **21** was synthesized from alcohol **15** in four steps. First, sugar **15** was transformed into 5-*O*-methanesulfonyl derivative **17**, followed by treatment with sodium azide to give compound **18** presenting a β-L-sorbopyranose configuration. Subsequent debenzoylation of 18 yielded azido alcohol **19**, which was finally subjected to hydrogenation with Pd/C to obtain amino alcohol ligand **21** (Scheme 1).

**Scheme 2:**
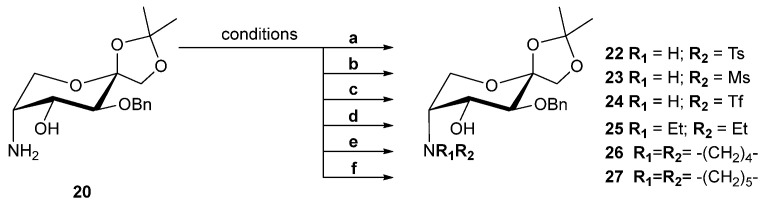
Synthesis of chiral β-aminoalcohol ligands derived from D-fructose. Reagents and conditions: (**a**) TsCl, Na_2_CO_3_, acetone-H_2_O; (**b**) MsCl, dry Py, 0 °C; (**c**) TfO_2_, Et_3_N, DMAP, dry DCM; (**d**) EtI, K_2_CO_3_, CH_3_CN, 60 °C; (**e**) 1,4-diiodobutane, K_2_CO_3_, CH_3_CN, 60 °C to 78 °C; and (**f**) 1,5-diiodopentane, K_2_CO_3_, 60 °C to 78 °C.

In order to synthesize the β-amino alcohol ligands with q D-fructopyranose structure (**22**–**27**), compound **20** was used as the starting material. β-hydroxysulfonamide ligands were first obtained through reaction of **20** with TsCl, MsCl, and TfO_2_, respectively, affording ligands **22**, **23**, and **24** in good yields. The dialkylated ligands (**25**–**27**) were obtained through a reaction of **20** with ethyl iodide, 1,4-diiodobutane, and 1,5-diiodopentane, respectively, to yield ligands **25**, **26**, and **27**, respectively (Scheme 2).

**Scheme 3:**
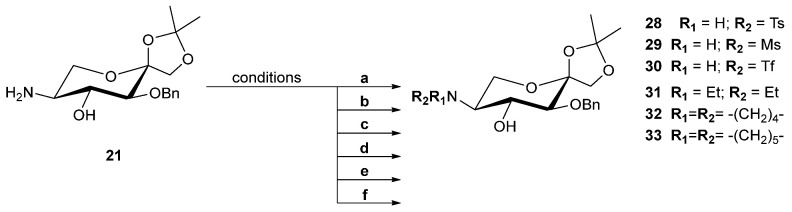
Synthesis of chiral β-amino alcohol ligands with L-sorbopyranose structures. Reagents and conditions: (**a**) TsCl, Na_2_CO_3_, acetone/H_2_O; (**b**) MsCl, dry Py, 0 °C; (**c**) Tf_2_O, Et_3_N, DMAP, dry DCM; (**d**) EtI, K_2_CO_3_, CH_3_CN, 60 °C; (**e**) 1,4-diiodobutane, K_2_CO_3_, CH_3_CN, 60 °C to 78 °C; and (**f**) 1,5-diiodopentane, K_2_CO_3_, 60 °C to 78 °C.

Ligands with L-sorbopyranose structure (**28**–**33**) were synthesized from compound **21** using the same procedure described above for ligands with D-fructopyranose structure (**22**–**27**) (Scheme 3).

Ligands **20** and **22**–**33** were then screened under the optimized conditions and their results are shown in Table 4. Conversion rates obtained with all ligands were good to excellent (78–100%). However, different levels of enantioselectivity were observed depending on the compound used. A dramatic loss of enantioselectivity was observed in *trans* β-aminoalcohols (**21** and **28**–**33**, entry 8–14). On the other hand, *cis* β-aminoalcohols (**20** and **22**–**27**, Table 4, entry 1–7) displayed higher enantioselectivity when compared with their *trans* counterparts. Importantly, all *cis* β-aminoalcohols (**20** and **22**–**27**, Table 4, entry 1–7) were able to catalyze the synthesis of the *S* enantiomer, while most of the *trans* β-aminoalcohols (**21**, **28**, **30**, and **32**, Table 4, entry 8, 9, 11, and 13) mainly deliver the *R* enantiomer.

Ligand **22** was the best compound evaluated (100% conversion and 92% *ee*) and was selected to explore the scope of substrates for this catalytic system. The enantioselective addition of Et_2_Zn in the presence of 20 mol % of ligand **22** (entry 2, Table 4) to a variety of aldehydes, such as aromatic, α,β-unsaturated, and aliphatic, both cyclic and linear were therefore studied (Table 5).

Ligand **22** proved to be catalytically active for all aldehydes tested, inducing high levels of conversion (70–100%). Moderate to good yields were also achieved for all aldehydes, the aliphatic substrates being the least reactive. In terms of stereoinduction, *o*- and *m*-methylbenzaldehydes showed excellent enantioselectivity (entries 2 and 3, 92% and 96% *ee*, respectively). These substrates slightly outperformed the enantioselectivity achieved for benzaldehyde (entry 1). As for the *p*-methylbenzaldehyde, the enantioselectivity decreased slightly (entry 4, 83% *ee*), indicating that the rate is influenced by the position of the methyl substituents on benzaldehyde. Good to excellent degrees of enantioselectivity were maintained in *p*-substituted benzaldehydes presenting both electron-donating and electron-withdrawing groups (entries 5, 6, and 7). With bulky aromatic aldehyde, 2-naphthaldehyde, the corresponding secondary alcohol was achieved with excellent enantioselectivity (entry 8, 92% *ee*). However, a lower level of enantioselectivity and moderate yield was achieved with the electron-rich heteroaromatic aldehyde, furfural (entry 9, 13% *ee*). Interestingly, in this substrate the secondary alcohol obtained was the *R*-enantiomer. A similar pattern of asymmetric induction was observed with cinnamaldehyde. This compound reacted with good yield and a moderate level of enantioselectivity (entry 9, 72% *ee*), giving the *R*-enantiomer of the corresponding secondary alcohol. Regarding the aliphatic aldehydes, Et*_2_*Zn addition generated the corresponding secondary alcohols with low enantioselectivity in the case of cyclic aliphatic, cyclohexanecarbaldehyde (entry 11, 35% *ee*), and moderate enantioselectivity for linear aliphatic hexanaldehyde (entry 12, 62% *ee*). It is worth mentioning the sense of enantioselectivity generated by ligand **22**. While most aldehydes tested underwent the ethyl group transfer to the Si face of the carbonyl group yielding the *S*-enantiomer, cinnamaldehyde and furfural (entries 8 and 9) showed the formation of the *R*-enantiomer. Such results could be attributed to the characteristic presence of the α,β-unsaturation in cinnamaldehyde and furfural causing a decrease in the energy difference between the two possible planar conformations in the active catalytic complex in both substrates, leading to a lower enantiomeric ratio [59].

## 3. Materials and Methods

Solutions were dried with MgSO_4_ before concentration under reduced pressure. The ^1^H and 13C NMR spectra were recorded with Variant INOVA UNITY 300 MHz, Variant DIRECT DRIVE 400 MHz, and 500 MHz spectrometers. Chemical shifts are quoted in ppm and are referenced to as residual H in the deuterated solvent as the internal standard. Splitting patterns are designated as follows: s, singlet; d, doublet; t, triplet; q, quartet; quint, quintet; m, multiplet, and br, broad. IR spectra were recorded with a Perkin–Elmer FT-IR Spectrum One instrument and mass spectra were recorded with Hewlett–Packard HP-5988-A and Fisons mod. Platform II and VG Autospec-Q mass spectrometers. Optical rotations were measured, unless otherwise stated, for solutions in CHCl_3_ (1 dm tube) with a Jasco DIP-370 polarimeter. TLC was performed on precoated silica gel (60 F254) aluminum sheets and compounds were detected with a mixture of H_2_SO_4_ in ethanol (5% in vol.), vanillin, KMnO_4_, and ammonium molybdate (10% *w*/*v*) in aqueous sulfuric acid (10%) containing cerium sulfate (0.8% *w*/*v*), and heating. Rf values refer to these TLC plates developed in the solvents indicated. Column chromatography was performed on silica gel silica gel 60 (230–400 mesh). The low-resolution mass spectra were recorded using an Agilent Technologies 1200 HPLC-MS system (6110 Quadrupole LC/MS) equipped with Zorbax Eclipse XDB-C18 columns (4.6 × 150 mm) and electrospray ionization (ESI). The high-resolution mass spectra were obtained using a High-Resolution Mass Spectrometer LCT-TOF Premier XE from Micromass Technology.

Enantiomeric excesses were determined by gas chromatography (GC) using a Hewlett-Packard 6890 chromatograph equipped with a flame ionization detector and chiral-fused silica capillary columns (30 m × 0.25 mm × 0.25 μm) from SUPELCO: Alpha-DEX™ 325, Beta-DEX™ 325, and Gamma-DEX™ 325, with helium as the carrier gas. The injector and detector temperatures were maintained at 270 °C.

Additionally, enantiomeric excesses were measured by high-performance liquid chromatography (HPLC) using an Agilent 1200 Series system coupled to a UV detector, with a CHIRALPAK IA chiral column from Daicel. **General procedure for enantioselective addition of diethylzinc aldehydes**.

In a Schlenk flask equipped with a magnetic stirrer (dried and under argon atmosphere) was added the ligand (20 mol%) which was then dissolved in hexane (0.25 mL), and Ti(OiPr)_4_ (104 µL, 0.35 mmol, 1.4 eq) was added and was kept under stirring at room temperature for 30 min. After this time, the reaction mixture was cooled to 0 °C, and 1 M solution of Et2Zn solution in hexane (0.75 mL, 0.75 mmol, 3 eq) was injected; the reaction mixture rapidly turned to a yellow color and was stirred for another 30 min at 0 °C. The reaction mixture acquired a dark green color, and we proceeded to the addition of the aldehyde (26.5 mg, 0.25 mmol) while stirring at a temperature of 0 °C for 3 h. After completion of the reaction, HCl 1N (3 mL) is added and then extracted with ether (3 × 5 mL). The organic phase was washed with water (3 × 5 mL) and dried with MgSO_4_. After filtration and concentration, the residue was purified by column chromatography (1:9/1:4, ether:hexane).

## 4. Conclusions

We successfully demonstrate the application of carbohydrate-derived chiral diol ligands based on D-glucose, D-galactose, and D-fructose, as well as the synthesis of novel amino alcohol ligands with D-fructose and L-sorbose structure, in the titanium-catalyzed enantioselective addition of diethylzinc to aldehydes. The glucose- and galactose-based diol ligands (**1**–**8**) exhibited promising catalytic activity, with several ligands achieving high conversions and moderate to high enantioselectivity. Among the fructose-derived ligands, those featuring a 1,2-*cis*-diol motif showed particularly strong performance, with ligand **9** reaching enantiomeric excesses of up to 72%.

Among the amino alcohol ligands, the one derived from D-fructose outperformed those based on L-sorbose. Most notably, ligand **22** exhibited the highest catalytic activity, affording high conversion rates and enantioselectivities of up to 98% *ee* across a broad range of aromatic and aliphatic aldehydes. The results obtained with **ligand 22** are comparable to, and in some cases surpass, those achieved with state-of-the-art systems. Ligand **22** is synthesized from an inexpensive, renewable monosaccharide (e.g., D-fructose) via a short, high-yielding synthetic sequence that avoids resolution steps and derivatization from non-renewable sources. Moreover, ligand **22** is air-stable, non-toxic, and structurally versatile due to its polyhydroxylated scaffold, offering a practical and sustainable approach for asymmetric catalysis.

This study reports, for the first time, the use of 5-amino-4-hydroxy pyranose monosaccharides as chiral ligands in catalytic asymmetric organometallic reactions. Furthermore, it highlights the utility of β-D-fructopyranose-configured monosaccharides as inexpensive, readily accessible, and tunable scaffolds for the development of highly enantioselective catalysts. Ongoing investigations aim to expand the substrate scope and evaluate the limitations of these ligand systems.

## Figures and Tables

**Table 2:** Evaluation of the catalytic activity of chiral diol ligands based on α-, β-D-methylglucopyranoside and α-, β-D-methylgalactopyranoside in the enantioselective addition of diethylzinc to benzaldehyde ^a^.

Ligand	Conversion (%) ^b^	*ee* (%) ^b^	Configuration ^c^
**1**	86	56	(*S*)
**2**	75	46	(*S*)
**3**	77	35	(*S*)
**4**	90	40	(*S*)
**5**	89	21	(*R*)
**6**	90	4	(*R*)
**7**	80	40	(*S*)
**8**	89	14	(*S*)

^a^ 0.25 mmol of benzaldehyde, 0.2 eq of ligand, 1.4 eq of Ti(O*^i^*Pr)_4_ and 3 eq of Et_2_Zn in 0.25 mL of hexane at 0 °C, 3 h. ^b^ Determined by GC using a Supelco α-DEX 325 column, helium flow: 0.8 mL/min, temperature: isothermal at 110 °C. ^c^ Absolute configuration assigned by comparing the optical rotation of the purified product with literature data.

**Table 3:** Evaluation of the catalytic activity of chiral diol ligands based on β-D-fructopyranose in the enantioselective addition of diethylzinc to benzaldehyde ^a^.

Ligand	Conversion (%) ^b^	*ee* (%) ^b^	Configuration ^c^
**9**	100	72	(*S*)
**10**	100	53	(*S*)
**11**	80	6	(*S*)
**12**	99	15	(*S*)
**13**	98	11	(*S*)
**14**	96	12	(*S*)

^a^ 0.25 mmol of benzaldehyde, 20 mol % of ligand, 1.4 eq of Ti(O*^i^*Pr)_4_, and 3 eq of Et_2_Zn in 0.25 mL of hexane at 0 °C, 3 h. ^b^ Determined by GC using a Supelco α-DEX 325 column, helium flow: 0.8 mL/min, temperature: isothermal at 110 °C. ^c^ Absolute configuration assigned by comparing the optical rotation of the purified product with literature data.

**Table 4:** Ligand screening in enantioselective addition of Et_2_Zn to benzaldehyde ^[a]^.

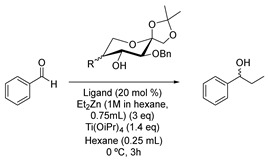						
Entry	Ligand	R	β-Amino Alcohol	Conversion ^[b]^	*ee* (%) ^[b]^	Configuration ^[c]^
1	**20**	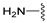	*E*	91	49	*S*
2	**22**	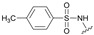	*E*	100	92	*S*
3	**23**	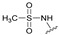	*E*	98	40	*S*
4	**24**	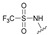	*E*	100	52	*S*
5	**25**	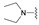	*E*	100	50	*S*
6	**26**	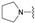	*E*	100	49	*S*
7	**27**	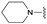	*E*	100	55	*S*
8	**21**	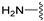	*Z*	78	2	*R*
9	**28**	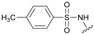	*Z*	91	30	*R*
10	**29**	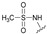	*Z*	98	1	*S*
11	**30**	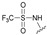	*Z*	100	4	*R*
12	**31**	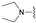	*Z*	100	3	*S*
13	**32**	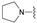 ^[c]^	*Z*	100	1	*R*
14	**33**	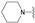 ^[c]^	*Z*	100	19	*S*

^a^ *Reaction conditions:* 0.25 mmol of benzaldehyde, 20 mol % of ligand, 1.4 eq of Ti(O*^i^*Pr)_4_, and 3 eq of Et_2_Zn in 0.25 mL of hexane at 0 °C. ^b^ Determined by GC using a chiral column Supelco α-DEX^TM^ 325, helium flow: 0.8 mL/min, and temperature: 110 °C. ^c^ Absolute configuration assigned by comparison with GC spectrum and the data described in the literature.

**Table 5:** Scope in enantioselective addition of Et_2_Zn to aldehydes ^[a]^.

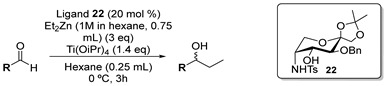					
Entry	R	Conversion (%) ^[b]^	Yield (%)	*ee* (%) ^[b]^	Configuration ^[c]^
1	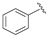	100	80	92	*S*
2	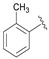	98	81	92	*S*
3	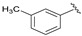	100	90	96	*S*
4	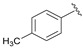	95	64	83	*S*
5	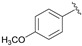	95	78	92	*S*
6	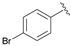	100	89	85	*S*
7	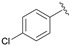	90	68	98	*S*
8	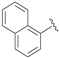	99 ^[e]^	84	92 ^[e]^	*S*
9	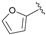	100	62	13	*R*
10	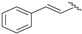	95	85	72	*R*
11	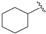	70	59	35	*S*
12	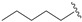	**81**	**57**	**62**	*S*

^a^ *Reaction conditions:* 0.25 mmol of aldehyde*,* 20 mol % of **3**, 1.4 eq of Ti(O*^i^*Pr)_4_, and 3 eq of Et_2_Zn in 0.25 mL of hexane at 0 °C. ^b^ Determined by GC. ^c^ Absolute configuration assigned by comparison with GC spectrum and data described in the literature. ^e^ Determined by HPLC.

## Data Availability

The original contributions presented in the study are included in the article, further inquiries can be directed to the corresponding authors.
